# Interdisciplinary Innovations and Applications of Bionics and Bioengineering in Kinesiology

**DOI:** 10.3390/bioengineering11101042

**Published:** 2024-10-18

**Authors:** Wei-Hsun Tai, Wenjian Wu, Haibin Yu, Rui Zhang

**Affiliations:** 1 School of Physical Education, Quanzhou Normal University, Quanzhou 362000, China; yhb@qztc.edu.cn; 2 Graduate School, Chengdu Sport University, Chengdu 610041, China; 3 School of Sports Science, Fujian Normal University, Fuzhou 350117, China; 4 Key Laboratory of Bionic Engineering Ministry of Education, China, Jilin University, Changchun 130022, China

Kinesiology, as an interdisciplinary field, emphasizes the study of human physical activity, with a particular focus on biomechanics and sports science. These disciplines provide essential insights into human movement mechanics, improving performance and facilitating rehabilitation by analyzing the forces involved in various physical activities [1]. Biomechanics, in particular, is critical for evaluating movement efficiency and injury prevention, providing valuable insights into how different physical activities impact the body’s musculoskeletal and neuromuscular systems [2,3]. Sports science complements biomechanics by applying physiological, psychological, and motor control theories to optimize athletic performance, enhance physical training methods, and promote recovery and rehabilitation [3,4]. The integration of these scientific approaches enables researchers and practitioners to address both theoretical and practical questions related to human movement, improving not only athletic outcomes but also health-related interventions for broader populations.

Despite its strengths, the field of kinesiology faces challenges due to its multidisciplinary nature, particularly in maintaining cohesion across the diverse research agendas of biomechanics, sports science, and related subfields. The specialized focus of these disciplines can lead to fragmentation within academic programs, with biomechanics often concentrating on technical analyses of movement, while sports science explores broader themes like exercise physiology and sport psychology [1,4]. To overcome these challenges, there is a growing trend toward interdisciplinary research, where biomechanics and sports science collaborate with fields like motor learning and exercise physiology to create comprehensive models of physical performance and well-being [4,5]. This integrated approach is advancing the understanding of complex human movements and contributing to the development of innovative training, rehabilitation, and performance enhancement techniques, positioning kinesiology as a key field in both academic and applied sports settings [6].

In recent years, kinesiology—the study of human movement—has experienced a transformative shift, driven by the interdisciplinary integration of bionics and bioengineering [7,8]. These fields, which combine biologically inspired systems with engineering principles, have brought about groundbreaking innovations in both sports performance and rehabilitation [9]. The convergence of these disciplines has not only enhanced our understanding of biomechanics but also expanded the scope of its applications in movement science. This Special Issue presents six pieces of cutting-edge research on the profound impact of this interdisciplinary approach, emphasizing the significance of bionic prosthetics [10], bioengineered footwear [11,12,13], and biomechanical modeling in optimizing human movement [14,15].

The first article, a bibliometric analysis by Shi et al., examines 1827 prosthetic foot studies from 2000 to 2022 [10]. The analysis reveals that the United States dominates this field, with Northwestern University being the leading institution. The study highlights four core research themes: demographics, functionality, psychology, and technology. Notably, diabetic-related amputations account for 75% of cases, underscoring the importance of prosthetic foot functionality for this population. Gait analysis emerges as a critical tool for evaluating prosthetic performance, while advancements in sensor integration and bionic design continue to drive innovation in the field. Li et al.’s study on the kinematics and kinetics of alternate foot jump-ropes compares barefoot and shod conditions [12], showing that footwear significantly reduces the MTP joint’s range of motion and enhances ankle joint propulsion. Their findings emphasize the importance of considering joint function in the design of jump-rope footwear to mitigate injury risk and improve performance.

The second group of studies delves into the biomechanics of specific athletic movements and equipment design. Wang et al. use a nine-segment biomechanical model to analyze how different body types affect balance support in gymnastics [15]. Their results indicate that individual anthropometrics play a pivotal role in performance, supporting the need for personalized training regimens. Zhou et al. employ finite element modeling to investigate the impact of different midsole structures on plantar pressure distribution in obese and healthy children [13]. The chiral structure offers superior pressure reduction, providing a theoretical foundation for an improved footwear design for overweight children. Casado-Hernández et al.’s cross-sectional study examines the effects of varying insole stiffness on plantar pressure in cycling, finding that stiffer insoles reduce pressure on metatarsal heads and toes, offering new insights into sports footwear design [11]. Finally, Agius et al. compare the kinematics of fixed versus sliding-seat rowing, demonstrating that fixed-seat rowing involves greater thoracic, pelvic, and shoulder movement, reducing the risk of back injury while emphasizing the biomechanical demands unique to this technique [14]. This Special Issue highlights the intersection of biomechanics and bionics in sports, showcasing the profound impact of these fields on performance enhancement, injury prevention, and the development of advanced athletic equipment. From prosthetic innovations to the biomechanical analysis of footwear and training techniques, the featured studies underscore the importance of personalized approaches in optimizing human movement.

Building on these foundational insights, we explore the interdisciplinary innovations and applications of bionics and bioengineering within kinesiology. These include the role of bionics in enhancing human performance and rehabilitation, as well as illustrating how these advancements bridge the gap between th biological limitations and technological possibilities. Moreover, we discuss bioengineering’s contributions to the design of footwear and athletic equipment, as well as how an understanding of biomechanics informs product development to ensure improved performance and injury prevention. Furthermore, we analyze how biomechanics and data-driven insights are reshaping movement science, demonstrating the collaborative potential of these fields in advancing our understanding of human movement and enhancing athletic training methodologies. These discussions underscore the significance of interdisciplinary approaches in driving innovation and improving outcomes in kinesiology.

## 1. The Role of Bionics in Enhancing Human Performance and Rehabilitation

Bionics, which applies the principles of biology to create technologies that mimic or augment natural human capabilities, has become a cornerstone of modern kinesiology. The development of bionic prosthetics and exoskeletons exemplifies the potential for these innovations to push the boundaries of human performance and rehabilitation. In particular, the creation of prosthetics that not only restore function but also exceed natural movement capabilities has enabled individuals with disabilities to perform tasks previously considered impossible. These devices integrate sensors, actuators, and robotics, allowing for precise, controlled movements that mirror natural biomechanics. For example, the study on bionic limbs and exoskeletons illustrates how these technologies are enabling athletes and individuals with physical impairments to perform tasks that surpass typical human capabilities. Exoskeletons designed for athletes reduce the metabolic cost of movement, allowing for sustained high-performance output [9]. The devices provide external support to the muscles and joints, minimizing fatigue and enhancing strength and endurance [16]. The implications for athletic training and competition are profound, as bionic technologies offer new ways to optimize performance, reduce injury risk, and accelerate recovery. In rehabilitation, bionics plays a crucial role in assisting patients with movement impairments [17]. Exoskeletons, for instance, are being used to facilitate motor retraining by providing real-time feedback on muscle activation and joint kinematics. This allows for more targeted and efficient rehabilitation protocols, leading to faster recovery times and improved movement outcomes [18,19,20]. As these technologies continue to evolve, they hold the potential to revolutionize both the prevention and treatment of musculoskeletal injuries in athletes and clinical populations alike.

## 2. Bioengineering’s Contributions to Footwear and Equipment Design

While bionics has made significant strides in augmenting human capabilities, bioengineering has focused on improving the tools and equipment used in both athletic and rehabilitative contexts. Footwear midsole design is a critical area in which bioengineering has had a notable impact [21]. In sports, the optimization of equipment—such as insoles for cycling—based on bioengineering principles has been shown to improve performance and comfort [22]. The effectiveness of hard insoles for plantar pressure in cycling, as demonstrated in a recent crossover study, highlights how customized, biomechanically optimized insoles can enhance force transfer and reduce discomfort during repetitive activities [11]. This research has implications for the design of footwear and other sports equipment, where bioengineering insights are being applied to create devices that support the body more effectively, reduce fatigue, and prevent overuse injuries. These innovations are not limited to professional athletes; they also hold significant promise for rehabilitation. Bioengineered devices like robotic exoskeletons and adaptive prosthetics are being designed to aid in the recovery process by providing support, stability, and real-time feedback during movement retraining [21]. The ability to customize these devices to an individual’s unique biomechanics ensures that rehabilitation is more effective and tailored to the patient’s specific needs [7]. While bioengineering focuses on optimizing tools and equipment, these innovations are deeply informed by biomechanical data, which provide critical insights into how such tools interact with human movement.

## 3. Biomechanics and Data-Driven Insights in Movement Science

At the heart of these advancements is the role of biomechanics in providing data-driven insights into human movement. The analysis of kinematics and kinetics under various conditions, such as barefoot versus shod jump-rope skipping, offers critical information on how different footwear choices influence joint mechanics and muscle activation patterns [12]. By employing one-dimensional statistical parameter mapping, researchers can accurately measure and compare movement dynamics under different conditions, guiding the design of performance-enhancing footwear and injury-reducing equipment.

Further, the development of advanced biomechanical models has allowed researchers to simulate complex human movements with remarkable precision. The study on the nine-segment biomechanical models used in gymnastics, for instance, demonstrates how anthropometric factors can influence the execution of movements such as the planche. By breaking down the body into distinct segments, researchers can analyze the forces and torques acting on each joint, providing valuable insights into how to optimize movement for both performance and safety [15]. These models are essential for designing individualized training regimens and bioengineered devices that align with an athlete’s specific physical characteristics, maximizing efficiency and minimizing injury risk.

## 4. Prosthetics and the Future of Bioengineering in Kinesiology

The intersection of bioengineering and prosthetics is one of the most promising areas of innovation in kinesiology. A scientometric analysis of prosthetic foot research from 2000 to 2022 reveals a growing trend towards integrating biomechanical data with bioengineered prosthetic designs [10]. Advances in materials, design, and real-time feedback mechanisms are allowing for the creation of prosthetics that adapt dynamically to different surfaces and movement conditions, offering greater functionality and comfort to users [18,19,20]. The interdisciplinary nature of this research highlights the importance of collaboration between bioengineers, biomechanists, and movement scientists. As the design of prosthetic limbs becomes more sophisticated, incorporating elements of biomechanics, robotics, and sensor technology, users will benefit from devices that not only restore function but also optimize it [3,17]. For example, prosthetic feet that adjust to changing terrain or wearable exoskeletons that provide additional strength and stability are blurring the line between human and machine, offering new possibilities for both athletic performance and rehabilitation [23,24]. Collaborative projects between bioengineers, sports scientists, and physiotherapists have resulted in breakthroughs such as prosthetic limbs with integrated sensors that adjust dynamically to terrain, improving both athletic performance and rehabilitation outcomes.

## 5. Conclusions

The integration of bionics, bioengineering, and kinesiology is revolutionizing human–machine systems, pushing beyond traditional human capabilities. Wearable sensors and exoskeletons are transforming both sports and rehabilitation by enhancing strength, endurance, and performance monitoring. As AI-driven models and machine learning optimize movement and recovery, kinesiology is entering a new phase in which biological and mechanical systems converge. This Editorial highlights these interdisciplinary breakthroughs, from footwear midsole design to advanced prosthetic technologies, underscoring their profound impact on movement science. The future of kinesiology will be defined by bioenhancement, where human abilities are augmented through technology, reshaping the limits of human performance and expanding the possibilities for training, recovery, and injury prevention.
